# Supplementary material for: A set of multi-entry identification keys to African frugivorous flies (Diptera, Tephritidae)
Source: Zookeys. 2014 Jul 24;(428):97–108. doi: 10.3897/zookeys.428.7366 (PMC4143993; doi:10.3897/zookeys.428.7366)
Supplement: Supplementary material 6 — Key to Ceratitis [file zookeys-428-097-s006.zip › SF6_ZooKeys_key to Ceratitis/key/SF6_ZooKeys_key to Ceratitis/Media/Html/Ceratitis malgassa.htm]

Ceratitis (Ceratitis) malgassa Munro


***Ceratitis*** **(*Ceratitis*) *malgassa*
Munro**

*Ceratitis* (*Ceratitis*) *malgassa*
Munro, 1939: 141.

Body
length: 5.06 (4.45-5.55) mm; wing length: 5.01 (4.65-5.55) mm.

Male

Head.
Antenna yellow or yellow-orange. Third antennal segment twice as long as second
segment. Arista almost bare, at most with very short hairs on base. Frons
yellow with orange patches; with short scattered hairs which are largely the
same colour as frons. Frontal and orbital bristles pale, upper orbital
sometimes dark, ocellar black; lower orbital modified, about as long as arista,
with apical end white and narrowly spatulate shaped, upper orbital and frontals
partly reduced, only moderately developed. Face yellow-white, lower eye margin
darker yellow. Genal bristle pale; genal setulae pale, weakly developed.
Postocellar and outer vertical pale.

Thorax.
Postpronotum yellow-white, with distinct black spot. Mesonotal pattern: ground
colour orange-brown, microtrichiae pattern silvery with brownish shine, spots
black-brown except rather indistinct sutural yellow or yellow-white spots,
prescutellar yellow markings merged. Scapular setae pale. Scutellum
yellow-white, basally with two dark narrowly merged spots, apically with three
merged spots, only slightly incised. Anepisternum with lower half darker yellow
but not distinct, hairs in lower half darker yellow. 

Legs
yellow; setation typical for subgenus, mainly pale especially on femora. Fore
femur posteriorly with bush of longer orange-coloured hairs along entire
length, posterodorsal hairs longer. Anteroventrally row of longer hairs, as
long as posterodorsal hairs; ventral spines yellow-orange. Mid femur
posteroventrally with row of longer hairs. Hind femur with longer hairs
dorsally and ventrally on apical fourth. 

Wing.
Banding; marginal band with narrow interruption, sometimes partly touching;
cubital band free; medial band joined with marginal band; crossvein r-m just
beyond middle of discal cell. Vein R1 before crossvein r-m. Orientation
crossvein dm-cu variable. 

Abdomen.
Yellow. Setation and banding typical for subgenus.

Female

As
in male except for the following characters:  Arista with slightly longer
hairs. Hairs on frons distinctly darker than frons. Darker colouration on lower
eye margin not outspoken. Frontal, orbital and ocellar bristles black, orbitals
not modified, frontal bristles well developed. Face sometimes darker. Genal
bristles dark or pale; genal setulae dark, strongly developed. Incisions of
apical scutellar spots not as deep as in male. Legs without feathering; ventral
spines on fore femur black. Oviscape shorter than abdominal terga.

(Description
after De Meyer, 2000)
